# Injection of Unicameral Bone Cysts with Bone Marrow Aspirate and Demineralized Bone Matrix Avoids Open Curettage and Bone Grafting in a Retrospective Cohort

**DOI:** 10.2174/1874325001711010486

**Published:** 2017-05-31

**Authors:** Kenneth R. Gundle, Etasha M. Bhatt, Stephanie E. Punt, Viviana Bompadre, Ernest U. Conrad

**Affiliations:** 1 Oregon Health & Science University, Department of Orthopaedics & Rehabilitation, Portland, USA; 2 Portland VA Medical Center, Operative Care Division, Portland, USA; 3 Department of Orthopaedics & Sports Medicine, University of Washington Medical Center, Seattle, Washington, USA; 4 Department of Orthopaedics & Sports Medicine, Seattle Children’s Hospital, Seattle, Washington, USA

**Keywords:** (Unicameral bone cyst, Bone marrow aspirate, Demineralized bone matrix, Less invasive surgery

## Abstract

**Background:**

Many treatment options exist for unicameral bone cysts (UBC), without clear evidence of superiority. Meta-analyses have been limited by small numbers of patients in specific anatomic and treatment subgroups. The purpose of this study was to report the outcomes of injecting bone marrow aspirate and demineralized bone matrix (BMA/DBM) for the treatment of proximal humerus UBC.

**Methods:**

Fifty-one patients with proximal humerus lesions treated by BMA/DBM injection were retrospectively reviewed from a single academic medical center.

**Results:**

The mean number of injections performed per patient was 2.14 (range 1-5). Eleven patients underwent only one injection (22%), an additional 19 patients completed treatment after two injections (37%), four patients healed after three injections (8%), and one patient healed after four injections (2%). The cumulative success rate of serial BMA/DBM injections was 22% (11/51), 58% (30/51), 67% (34/51), and 69% (35/51). Eleven patients (22%) ultimately underwent open curettage and bone grafting, and five patients (10%) were treated with injection of calcium phosphate bone substitute.

**Conclusion:**

A BMA/DBM injection strategy avoided an open procedure in 78% of patients with a proximal humerus UBC. The majority of patients underwent at least 2 injection treatments.

**Level of Evidence:**

Level IV retrospective cohort study.

## INTRODUCTION

Unicameral bone cysts (UBCs), also known as simple bone cysts, are benign bone tumors that most commonly present in childhood and adolescence [1]. The most common areas for these lesions to occur are the metaphysis of the proximal humerus (50%) and the proximal femur (18-27%) [2]. Typically, they are discovered incidentally or after a fracture.

Many treatment methods exist for treating UBCs, including observation, steroid injection [3-5], bone marrow injection [6-9], curettage and bone grafting [10-12], or intramedullary drilling with or without nailing [13-16]. Indications for treatment include ongoing pain and the prevention of pathologic fracture, with conservative treatment of symptomatic lesions resulting in low rates of success [17].

A recent systematic review and meta-analysis assessed the results of 497 humerus UBCs treated by 16 different modalities [17]. Injection with bone marrow and demineralized bone matrix (BMA/DBM) was successful in 100% (28/28) patients treated, comparing favorably with only a 22% (12/53) success rate with conservative treatment, and 81% (86/106) success rate with steroid injection [17]. Due to the small sample of patients in the BMA/DBM subgroup (28/497 or 5.6%), however, there was insufficient evidence to recommend BMA/DBM over steroid injection or a more invasive open curettage. Therefore, further data on the outcomes of this treatment modality would aid subsequent meta-analyses and guide potential trials of less invasive injection treatment versus open curettage.

The study sought to answer the following questions: What is the rate of repeat operation after a single treatment with BMA/DBM? How many treatments with BMA/DBM do patients undergo? How often does a patient proceed to open curettage and bone grafting after treatment with BMA/DBM?

## MATERIALS AND METHODS

The records of a single orthopaedic oncologist at a tertiary pediatric hospital were retrospectively reviewed for the extraction of demographic and treatment data. This study received institutional review board approval. Surgical case logs were cross-referenced with surgical calendar schedules to facilitate the identification of all subjects. Between 1980 and 2013, 217 patients with a UBC that underwent surgical treatment were identifiedfor the senior author (EUC) indications include ongoing pain, preventing pathological fracture, cortical thinning on x-ray or cyst larger than 3 cm. Patients were identified through January of 2013, to allow at least 12 months of follow-up and any reoperations through January of 2014. Only patients with a UBC in the proximal humerus and whose initial treatment was BMA/DBM were included. In 51% (110/217) of patients, the UBC location was the proximal humerus and 46% (51/110) of those patients were treated with BMA/DBM injection as the primary treatment.

The treatment evaluated in this study was the injection of UBCs with BMA/DBM. The operative protocol consisted of advancing a #10 or 3mm Jamshidi needle under fluoroscopic control into the cyst, and injecting radiopaque dye to confirm the cystic nature of the lesion. Ten milliliters of autogenous marrow was aspirated from the ipsilateral anterior superior iliac spine using a Jamshidi needle and was mixed with 10 mL of demineralized bone matrix. The cyst was then injected with 10 mL of the combined BMA/DBM *via* a 20 mL syringe. Patients were discharged the same day with a sling for comfort and advanced activities as tolerated. Follow-up included clinical examination and routine radiographs at six weeks and three months post operatively, with additional follow-up if symptoms continued or a cyst larger than 3cm persisted.

The indication for initial treatment as well as repeat treatment was fracture, humeral pain, or cysts larger than 3 cm on x-ray with cortical thinning. The primary endpoint was progression to additional surgical treatment-either repeat injection with BMA/DBM or open curettage and bone grafting. Bone cyst healing after treatment was defined as an asymptomatic cystic defect smaller than 1.0cm. This definition of healing is consistent with previous studies, which have noted the need to treat only lesions at risk for pain or fractures [10, 16]. Decision making regarding additional treatment was made at least 3-6 months after the procedure.

Statistical analysis was completed with Stata version 11 (College Park, Texas). Continuous variables were compared with a two-tailed t-test, with alpha set at the 0.05 level.

## RESULTS

All 51 patients in this cohort were included in the analysis (see Table **1**). Mean length of follow-up was 34 months (range 2-96 months). Twelve subjects had their final follow-up visit less than one year post-operatively, though at least one year had transpired between the operation and the end of the study period. The mean age at the first treatment was 10.3 years (range 3.4-18 years, SD 3.4). There were 11 females and 40 males. There was no difference in the reoperation rate by gender (p=0.85), nor was there a correlation between age and the number of operations (p=0.84). This male to female ratio is similar to prior studies of UBC.

**Fig. (2):**
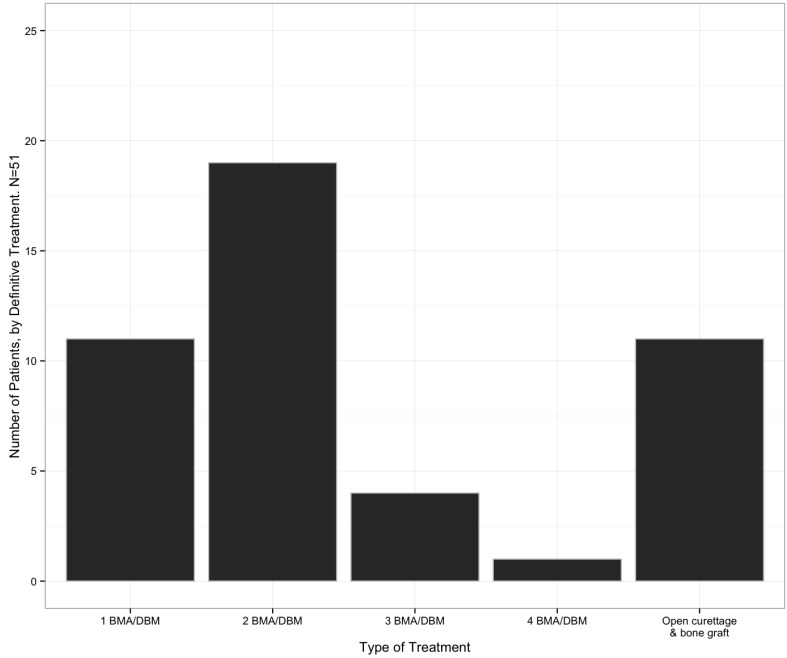
Number of patients with proximal humerus unicameral bone cysts that healed after each treatment.  BMA/DBM: Bone marrow aspirate and demineralized bone matrix injection.

The mean number of injections performed per patient was 2.14 (range 1 to 5, Fig. (**1**)). Out of the 51 patients initially treated with BMA/DBM, 22% (11/51) received no further operative treatment. Forty patients returned for a second procedure, at this point, 32 received injections and 19 of these were successful (59%). After one injection, at the senior author’s discretion, six patients instead received an open curettage and allograft, and one of these patients later underwent a repeat curettage and allograft procedure. Two patients underwent injection with a calcium phosphate bone graft substitute, and both healed without further operative treatment. After the second injection, 13 patients returned for a third procedure. After three patients were removed from the injection pathway for open curettage and bone grafting and three underwent injection with calcium phosphate, a total of seven patients received a third injection with BMA/DBM. Four of the seven patients (57%) required no additional treatments. Of the remaining three patients, one had surgical curettage and allograft bone grafting, and two had a fourth injection with BMA/DBM. After the fourth BMA/DBM treatment, one healed and one later required surgery with open curettage and allograft bone. The cumulative success of the BMA/DBM injection strategy over four rounds of injections was 22%, 59%, 67%, and 69%, respectively. Healing was achieved in 11 patients (22%) after one injection, 19 patients (37%) after two injections, 4 patients (8%) after three injections, and one patient (2%) after four patients (Fig. **2**).

Eleven of 51 patients (22%) ultimately progressed to open curettage and bone grafting and one of these required a subsequent procedure. Additionally, five patients (10%) underwent injection with calcium phosphate bone graft substitute, and none of these cases underwent further operative treatment.

## DISCUSSION

This retrospective study of 51 patients treated with BMA/DBM injection for UBC of the proximal humerus showed an average of over two injections per patient, with only 22% of patients healed with a single injection. There were few successful treatments after a course of three injections, but overall this strategy of BMA/DBM only transitioned to open curettage and bone grafting in 11 of 51 patients (22%). This patient population almost doubles the overall number of proximal humerus UBCs treated with BMA/DBM reported in a recent meta-analysis [17].

The present study found that just over 20% of patients underwent a single injection with BMA/DBM without further treatment. This is notable lower than the 59% rate of first BMA/DBM injection healing reported by Bella *et al.* [18], though most reports contain UBCs from multiple locations and some have noted higher rates of retreatment in the humerus [19]. Kanellopoulos **et al.*.* [7] report a combination of percutaneous reaming followed by injection of demineralized bone matrix and autologous bone marrow, with 89% (17/19) receiving only one. Their higher rate of single treatment success may be attributable to the inclusion of percutaneous reaming and restoration of medullary continuity, as this may resolve the proposed etiologic venous obstruction while also allowing native bone marrow to enter the cyst [20].

Although the single injection success rate was low, 59% of patients healed after two BMA/DBM treatments, and 67% were cumulatively successful after three injections. The marginal gain in cumulative success after three injections, from 67% to 69%, suggests diminishing returns of this strategy after three attempts. Detailed information on the course of treatment may aid in patient and parent education if considering UBC treatment with BMA/DBM injections.

An advantage of treatment with injections is that they are less invasive than open procedures. The endpoint for the current study was the decision to convert from injection of BMA/DBM to open curettage and bone grafting. The treatment philosophy of the senior author (EUC) is to attempt three such injections before resorting to open treatment. An injection-based treatment failure rate of 19% reported by Bella **et al.*.* [8] is consistent with the present study, in which 22% (11/51) of patients were eventually treated with curettage and bone grafting after an unsuccessful course of injections.

Several limitations may affect the generalizability of these results. The retrospective design and potential for selection bias may falsely overstate the success of the treatment method. There is also little consensus about indications for treatment or definitions for healing after treatment. For the senior author (EUC) indications include ongoing pain, preventing pathological fracture, cortical thinning on x-ray or cyst larger than 3 cm. The criteria for healed UBC were an asymptomatic lucency smaller than 1 cm on x-ray. The decision for a second injection was made at least 3-6 months after the first injection. It is possible that more UBCs may have healed after a single injection with additional conservative treatment, or may have healed with an additional injection rather than converting to open curettage. Differential indication for repeat operation may explain the higher rate of repeat injection in the present study.

Another potential limitation is that not all patients received a course of three injections before considering an alternative treatment. Six patients underwent open curettage and bone grafting after a single injection, and three patients transitioned to open curettage after just two injections. It is possible that injections may have succeeded in these cases with additional follow-up, which would have improved the success of a BMA/DBM injection strategy and further reduced the rate of conversion to a more invasive procedure. Of note, five patients were treated with calcium phosphate injection as a bone graft substitute, without need for subsequent surgical treatment. This result is similar to Thawrani *et al.*, who reported that 13 of 13 patients injected with a form of calcium phosphate where asymptomatic without further procedures at final follow-up [21]. The small number of such patients in this series, and lack of specific algorithm for its use during the study period, limits analysis of this treatment strategy. A trial comparing BMA/DBM with calcium phosphate injection may be warranted.

Treatment of symptomatic UBCs of the proximal humerus with BMA/DBM is a reliable means of avoiding the need for a larger surgery such as open curettage and bone grafting. However, patients and families should be counseled on the potential need for more than one injection to obtain an effective result. Future comparative studies on the different injection methods might be useful in guiding clinical decision making, as would the assumption of some consensus regarding indications and the radiographic “healing” of unicameral bone cysts after treatment.

## Figures and Tables

**Fig. (1):**
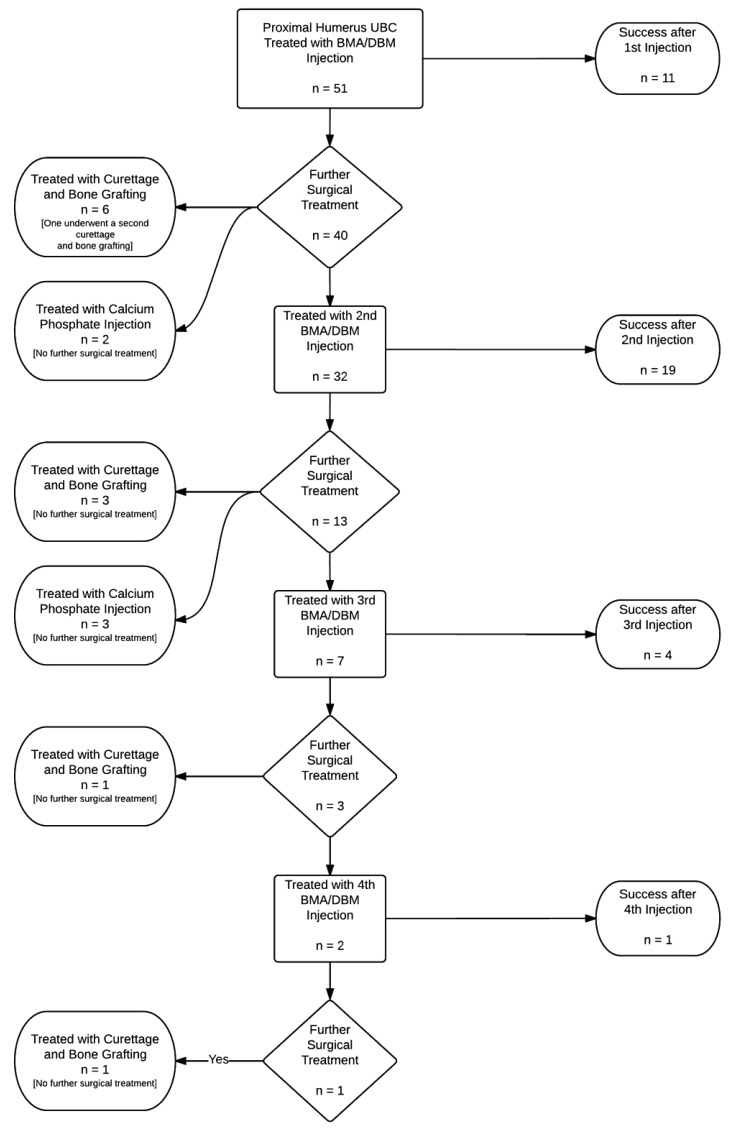
Flowchart of study patients with proximal humerus unicameral bone cysts initially treated with an injection of bone marrow aspirate with demineralized bone matrix.

**Table 1:** Repartition of groups according to type of contention.

n	51
Female (n, %)	11 (22%)
Age (years)	10,Range 3.4 to 18
Follow-up (months)	34,Range 2-96
Number of surgeries	Mean 2,SD 0.9, Range 1-5

## LIST OF ABBREVIATIONS

BMA/DBM: = Bone marrow aspirate with demineralized bone matrix
UBC: = Unicameral bone cyst

## References

[1] Mirra J.M., Picci P., Gold R.H. (1989). Bone tumors..

[2] Herring J. (2007). Benign musculoskeletal tumors.. Tachdjian's pediatric orthopaedics.

[3] Scaglietti O., Marchetti P.G., Bartolozzi P. (1979). The effects of methylprednisolone acetate in the treatment of bone cysts. Results of three years follow-up.. J. Bone Joint Surg. Br..

[4] Scaglietti O., Marchetti P.G., Bartolozzi P. (1982). Final results obtained in the treatment of bone cysts with methylprednisolone acetate (depo-medrol) and a discussion of results achieved in other bone lesions.. Clin. Orthop. Relat. Res..

[5] Capanna R., Dal Monte A., Gitelis S., Campanacci M. (1982). The natural history of unicameral bone cyst after steroid injection.. Clin. Orthop. Relat. Res..

[6] Lokiec F., Ezra E., Khermosh O., Wientroub S. (1996). Simple bone cysts treated by percutaneous autologous marrow grafting. A preliminary report.. J. Bone Joint Surg. Br..

[7] Kanellopoulos A.D., Yiannakopoulos C.K., Soucacos P.N. (2005). Percutaneous reaming of simple bone cysts in children followed by injection of demineralized bone matrix and autologous bone marrow.. J. Pediatr. Orthop..

[8] Di Bella C., Dozza B., Frisoni T., Cevolani L., Donati D. (2010). Injection of demineralized bone matrix with bone marrow concentrate improves healing in unicameral bone cyst.. Clin. Orthop. Relat. Res..

[9] Docquier P-L., Delloye C. (2003). Treatment of simple bone cysts with aspiration and a single bone marrow injection.. J. Pediatr. Orthop..

[10] Neer C.S., Francis K.C., Johnston A.D., Kiernan H.A. (1973). Current concepts on the treatment of solitary unicameral bone cyst.. Clin. Orthop. Relat. Res..

[11] Hagmann S., Eichhorn F., Moradi B., Gotterbarm T., Dreher T., Lehner B., Zeifang F. (2011). Mid- and long-term clinical results of surgical therapy in unicameral bone cysts.. BMC Musculoskelet. Disord..

[12] Schreuder H.W., Conrad E.U., Bruckner J.D., Howlett A.T., Sorensen L.S. (1997). Treatment of simple bone cysts in children with curettage and cryosurgery.. J. Pediatr. Orthop..

[13] Roposch A., Saraph V., Linhart W.E. (2000). Flexible intramedullary nailing for the treatment of unicameral bone cysts in long bones.. J. Bone Joint Surg. Am..

[14] Hou H-Y., Wu K., Wang C-T., Chang S.M., Lin W.H., Yang R.S. (2011). Treatment of unicameral bone cyst: surgical technique.. J. Bone Joint Surg. Am..

[15] de Sanctis N., Andreacchio A. (2006). Elastic stable intramedullary nailing is the best treatment of unicameral bone cysts of the long bones in children?: Prospective long-term follow-up study.. J. Pediatr. Orthop..

[16] Kanellopoulos A.D., Mavrogenis A.F., Papagelopoulos P.J., Soucacos P.N. (2007). Elastic intramedullary nailing and DBM-bone marrow injection for the treatment of simple bone cysts.. World J. Surg. Oncol..

[17] Kadhim M., Thacker M., Kadhim A., Holmes L. (2014). Treatment of unicameral bone cyst: Systematic review and meta analysis.. J. Child. Orthop..

[18] Sung A.D., Anderson M.E., Zurakowski D., Hornicek F.J., Gebhardt M.C. (2008). Unicameral bone cyst: A retrospective study of three surgical treatments.. Clin. Orthop. Relat. Res..

[19] Rougraff B.T., Kling T.J. (2002). Treatment of active unicameral bone cysts with percutaneous injection of demineralized bone matrix and autogenous bone marrow.. J. Bone Joint Surg. Am..

[20] Givon U., Sher-Lurie N., Schindler A., Ganel A. (2004). Titanium elastic naila useful instrument for the treatment of simple bone cyst.. J. Pediatr. Orthop..

[21] Thawrani D., Thai C.C., Welch R.D., Copley L., Johnston C.E. (2009). Successful treatment of unicameral bone cyst by single percutaneous injection of alpha-BSM.. J. Pediatr. Orthop..

